# Biochemical and Pharmacological Studies on Kynurenic Acid Metabolism in the *Helix pomatia*—Snail Model of Learning and Memory

**DOI:** 10.3390/biom16040603

**Published:** 2026-04-18

**Authors:** Halina Baran, Carina Kronsteiner

**Affiliations:** 1 Karl Landsteiner Research Institute for Neurochemistry, Neuropharmacology, Neurorehabilitation and Pain Treatment Mauer, Neuropsychiatric Hospital Mauer, Hausmenninger Straße 221, 3362 Mauer bei Amstetten, Austria; 2 Department of Biomedical Sciences, University of Veterinary Medicine Vienna, 1210 Vienna, Austria

**Keywords:** *Helix pomatia*, snail model of learning and memory, tentacle lowering, kynurenic acid, KAT, CNS, ganglia, liver, heart, L-kynurenine, Cerebrolysin, D-cycloserine, NMDA receptor, nAChR, GPR-35

## Abstract

Kynurenic acid (KYNA), a metabolite of the L-kynurenine pathway of L-tryptophan degradation, is an endogenous blocker of glutamate ionotropic excitatory amino acid (EAA) receptors and nicotinic acetylcholine receptors (nAChRs). KYNA plays a significant role in various neuropsychiatric disorders and the aging process. Some researchers have suggested that KYNA may contribute to memory impairment. In this study, we examined the impact of L-kynurenine (a KYNA substrate) and the anti-dementia drugs D-cycloserine and Cerebrolysin on kynurenine aminotransferase (KAT) activity, an enzyme forming KYNA, in liver homogenates of *Helix pomatia* snails. Furthermore, a memory model was established using these snails, wherein tentacle shortening served as an indicator of learning activity. In vitro experiments on *Helix pomatia* demonstrated the significant impact of L-kynurenine and anti-dementia drugs on KYNA synthesis. KYNA levels increased significantly in the presence of L-kynurenine in liver homogenate. However, KYNA formation decreased when anti-dementia drugs, including Cerebrolysin or D-cycloserine, were administered to the snails’ liver homogenate. L-kynurenine has been shown to impair the learning process in vivo in snails, but an anti-dementia drug has been demonstrated to reverse this effect. Significant inhibition of tentacle lowering was observed in response to L-kynurenine treatment, which corresponded with elevated KYNA levels in the central nervous system. Administering D-cycloserine or Cerebrolysin alongside L-kynurenine reversed its effects. The *Helix pomatia* memory model is a valuable tool for studying learning and memory formation in various conditions and in the presence of different pharmacological agents. A drug or natural extract that blocks KYNA synthesis has the ability to increase tentacle lowering and could be considered an anti-dementia agent. Furthermore, this metabolite may also protect against aging and delay damage to the central nervous system related to memory.

## 1. Introduction

**Figure 1:**
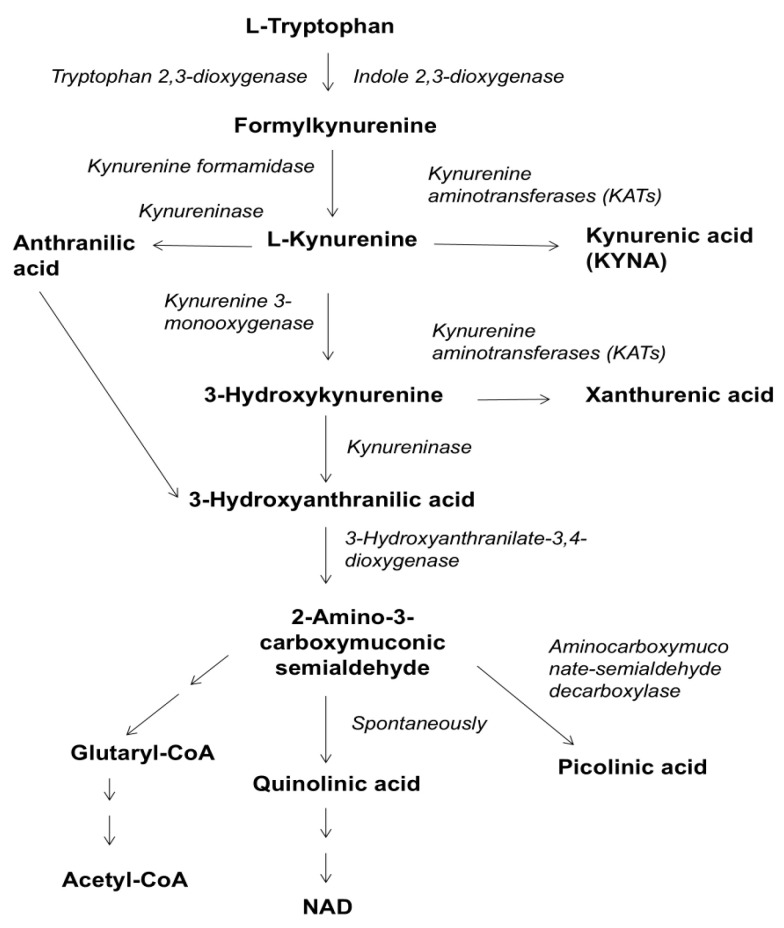
Kynurenine pathway of tryptophan metabolism.

As previously demonstrated in 1996 and in 1999, increased kynurenic acid (KYNA) formation was found in the brain of patients with Alzheimer’s disease (AD) [1]. This finding has since been confirmed in later studies [2,3,4,5,6,7]. A substantial body of research observed the correlation between elevated KYNA metabolism and memory impairment [7,8,9,10,11]. Some studies have reported an association between lower levels of KYNA in the blood of Alzheimer’s patients and dementia. Hartai et al. demonstrated lower KYNA levels in the serum of Alzheimer’s patients [12]. Gulaj et al. found this to be the case too [13]. We have also observed reduced levels of KYNA in the blood and cerebrospinal fluid (CSF) of Alzheimer’s patients [1]. Nevertheless, we believe that increased KYNA synthesis in the brain provides a more convincing explanation for memory impairment. Zwilling et al. (2011) presented an interesting pharmacological approach involving the use of a kynurenine 3-monooxygenase inhibitor (see Figure 1) to increase KYNA levels in the blood of transgenic mice with Alzheimer’s disease [14]. The authors observed the prevention of spatial memory deficits, anxiety-related behaviors, and synaptic changes. They also observed decreased microglia activation in a mouse model of Huntington’s disease. The data reveal significant differences in the mechanisms presented. Further clarification of the diagnosis and disease characteristics through biochemical investigations is required, as well as consideration of disease duration, age, and species.

KYNA, a metabolite of tryptophan degradation along the kynurenine pathway (see Figure 1), acts as an endogenous blocker of glutamate ionotropic excitatory amino acid receptors: N-methyl-D-aspartate (NMDA) receptors [15,16] and the α7 nicotinic acetylcholine receptor (α7 nAChR) subtype [17]. KYNA is also an agonist at the orphan G protein-coupled receptor GPR35 [18,19] and has been suggested to exhibit neuroprotective and anticonvulsant properties [20,21]. At physiological concentrations ranging from low nanomolar to low micromolar, KYNA is likely to block NMDA receptors [15,16] and the α7 nAChR subtype [22,23].

Greenmayer et al.’s hypothesis, first proposed in 1988 and still supported today, is that glutamatergic neurotransmission plays an important role in the symptoms of dementia [24]. We recently discussed the significance of KYNA in various neuropsychiatric disorders and concluded that increased KYNA levels in the central nervous system (CNS) may contribute to cognitive decline [25]. Increased KYNA levels in the CNS have also been observed in healthy elderly individuals [26]. Alongside the proposed roles of glutamate [15,16] and KYNA-mediated blockade of glutamate and/or acetylcholine receptors [15,16,17], these findings suggest the potential therapeutic value of the L-kynurenine pathway in treating cognitive and/or learning impairments.

Learning ability naturally declines with age and is associated with neurodegenerative processes that are characterized by accompanying gliaproliferation, and by increased KYNA metabolism in the CNS [25]. Knowledge of KYNA action as an antagonist of the ionotropic excitatory glutamate and α7 nAChR subtypes is documented [15,16,17]. However, controversy remains regarding KYNA’s actions on the α7 nAChR subtype. In 2020, Stone suggested that there is no reliable evidence of KYNA’s blocking activity at nAChRs [27]. Furthermore, Stone recommended using only information regarding the blocking effect on glutamatergic neurons [27]. However, accumulated data indicate that KYNA affects both cholinergic and glutamatergic receptors [16,22,23].

KYNA is formed from L-kynurenine via the action of kynurenine aminotransferases (KATs). Initially identified in the periphery, KATs were subsequently found in the CNS of mammals [28,29,30]. In previous work, my co-worker, Kepplinger, and I demonstrated that Cerebrolysin and D-cycloserine reduce KYNA formation by inhibiting enzymes involved in KYNA synthesis, including KATs [31,32]. Furthermore, the presence of meat or herbs in a KYNA assay in vitro also impedes KAT activity [33,34,35]. Analysis of accumulated data suggests that dietary restriction may affect KYNA levels in the CNS. Indeed, dietary restriction has been shown to activate NMDARs in critical interneurons involved in learning [36]. Consistent with this finding, Potter et al. demonstrate that reducing endogenous KYNA synthesis increases extracellular levels of glutamate, enhancing plasticity in the brain (e.g., the hippocampus) and, notably, cognitive behavior [37].

Historically, snails have been used as a food supplement [38]. Furthermore, they have gained prominence in scientific research focused on the study of learning processes, as highlighted by Acebes et al. in 2012 [39]. Their relatively simple systems make them ideal subjects for studying basic levels [40].

Scientists have demonstrated that invertebrate species, such as the *Helix aspersa* snail, can learn to associate a stimulus (CS) with food (US) [41]. In the context of learning, ‘tentacle lowering’ refers to a specific, robust appetitive Pavlovian conditioning procedure used to investigate memory and associative learning in snails, as demonstrated by Muñiz-Moreno and Loy (2022) in *Helix aspersa* [42]. The authors demonstrated how snails learn to associate an odor with food. This results in an increased response of tentacle lowering when the odor is presented alone. Increased levels of tentacle lowering in the presence of the stimulus indicate successful learning and memory formation. This response serves as a measure of cognitive function, including learning, memory, attention, alertness, and complex cognition [42]. Increased tentacle lowering in the presence of the stimulus indicates successful learning and memory formation. This response can be used to measure cognitive function in terms of learning and memory, attention, alertness, and complex cognition [42].

The snail *Helix pomatia* synthesizes KYNA in the central and peripheral nervous systems [43]. Given its presence in the mammalian brain [44] and its regulation by the blood–brain barrier [45], snails could be valuable models for studying the link between KYNA synthesis and memory impairment. This phenomenon may be due to the relatively underdeveloped blood–brain barrier in these subjects, which allows pharmaceutical agents to permeate the ganglia [39,40,45].

This study aims to investigate how L-kynurenine metabolism in the liver of the *Helix pomatia* snail is altered by various pharmacological treatments, including L-kynurenine, Cerebrolysin, and D-cycloserine. The influence of these treatments on the snail’s tentacle reduction response is of interest, as are the effects on attention and alertness. Finally, the study investigated alterations in KYNA levels in the brain homogenate of the snail. This evaluation included an assessment of learning and memory capacity.

## 2. Materials and Methods

### 2.1. Compounds

The following compounds: L-kynurenine; KYNA; pyruvate; pyridoxal-5′-phosphate; 2-amino-2-methyl-1-propanol (AMPOL); and D-cycloserine were purchased from Sigma–Aldrich Handel’s GmbH, Vienna, Austria. Cerebrolysin was a gift from EBEWE–Pharma Ges.mbH Nfg.KG (Mondseestraße 11, 4866–Unterach am Attersee, Austria). Cerebrolisin is produced by standardized, controlled enzymatic digestion of lipid-free porcine brain proteins and consists of free amino acids and peptides with molecular weights of less than 10 kD. In solution, it contains 40 mg of dry substance per mL with a nitrogen content of 5.3 mg. All other chemicals used were of the highest commercial purity and were obtained from VWR International GmbH, BDH Chemicals, Vienna, Austria, and from Merck KGaA 64271–Darmstadt, Germany.

### 2.2. Solutions for In Vivo Pharmacological Experiments

L-kynurenine is dissolved in the feed water to achieve a final concentration of 61.3 mg/mL. The pH is then adjusted to a neutral pH of 7 using 2N and 4N KOH. D-cycloserine is dissolved in the feed water to a final concentration of 5.1 mg/mL. Cerebrolysin is dissolved in make-up water to a final concentration of 176.5 µL/mL. A mixture of the L-kynurenine/D-cycloserine and L-kynurenine/Cerebrolysin solutions is prepared by dissolving them in water at the same concentrations as above. Adjust the solutions to a pH of 7 by adding 2N and 4N KOH.

### 2.3. Animals-Snails

*Helix pomatia* snails, obtained from the Schnecke Manufactory Gugumuck (Rosiwalgasse 44, 1100 Vienna, Austria), were housed in large groups in an indoor enclosure with access to vegetables (lettuce and carrots) and a 12-h light/dark cycle. The enclosure was watered to maintain optimal humidity. The study was performed at the Karl Landsteiner Research Institute in Mauer, NÖ, Austria.

### 2.4. Age Determination of Snails ARS- Middle-Aged Group of Snails

Age was determined using an Age Rating Scale (ARS), consisting of measurements of several parameters recently described by Kronsteiner et al. (2023) [43]. This study used middle-aged snails with a body ranging from 9 to 16 g.

### 2.5. Preparation of Region and Homogenates

Once the experiments were complete, the snails were killed by freezing them at −45 °C. They were then stored until they were analyzed. For dissection, the snails were thawed, and their livers and nerve rings were removed [43]. The tissue was used to determine the parameters.

Homogenate for KATs: tissue samples (liver or ganglia) were homogenized in an ice bath using a ratio of five volumes of 5 mM Tris-acetate buffer solution (pH 8.0) containing 50 µM pyridoxal 5′-phosphate and 10 mM mercapto-ethanol (wt/vol). These homogenates were then used to determine the activities of KAT I, KAT II, and KAT III. Formed KYNA was determined using high-performance liquid chromatography (HPLC).

Homogenate for KYNA, tissue (liver or brain) homogenates were prepared by adding 19 or 5 volumes (wt/vol) of distilled water, respectively, homogenizing the mixture, and dividing it into two portions: one for measuring KYNA levels and one for measuring KAT activity, if necessary. For KYNA measurements, the homogenates were mixed with 50% trichloroacetic acid (*v*/*v*). Denatured proteins were then removed via centrifugation at 11,700 rpm for 20 min at 7 °C. KYNA was determined using HPLC.

### 2.6. Biochemical Determination Method

#### 2.6.1. KAT Assay

KAT activities were determined as previously described by Baran et al. (1999) [1]. Briefly, the reaction mixture contains: a homogenate, 2 µM L-kynurenine, 1 mM pyruvate, 70 µM pyridoxal 5′-phosphate, and buffer: for KAT I (150 mM AMPOL buffer, pH 9.6); for KAT II (150 mM Tris-acetate buffer, pH 7.4); and for KAT III (150 mM Tris-acetate buffer, pH 8.0). After two hours of incubation at 37 °C (pilot experiments demonstrated the linearity of enzyme activity up to 18 h), we terminated the reaction by adding 10 µL of 50% trichloroacetic acid (TCA). We added 1 mL of 0.1 M HCl and centrifuged for 10 min to remove denatured proteins. If necessary, we applied the supernatant to a DOWEX 50 W anion exchange column, as described by Turski et al. (1989) [46]. KYNA eluted from the column was determined using HPLC. Blanks were obtained by adding 10 µL of 50% TCA prior to incubation at 37 °C.

#### 2.6.2. KYNA Measurement

KYNA measurements were performed according to the described method [47], with modifications [1]. The mobile phase consisted of 50 mM sodium acetate, 250 mM zinc acetate, and 4% acetonitrile (pH 6.15). The mobile phase was pumped through a 10 cm × 0.4 cm HR-80 C-18 column (3 µm particle size, In Chrom, Vienna, Austria) at a flow rate of 1.0 mL/min. The fluorescence detector L-2485 Hitachi was set to excitation and emission wavelengths of 340 and 398 nm, respectively. The injection volume was 50 µL. KYNA had a retention time of approximately seven minutes, with a sensitivity of 50 fmol per injection and a signal-to-noise ratio of 5. The HPLC system consisted of the following components, sourced from VWR Hitachi, Vienna, Austria: Elite Lachrom: Organizer; Pump L-2130; Column Oven L-2300; Fluorescence Detector L-2485; Autosampler L-2200, and Integrator HP Compaq and Drucker HP LaserJet P1006, Vienna, Austria.

## 3. Pharmacological Study—In Vitro

### 3.1. Synthesis of KYNA in Snail Liver Homogenate

To verify the synthesis of KYNA in snail liver homogenate (1:100, *w*/*v*), the reaction mixture was incubated in the presence of various concentrations of L-kynurenine (50 µM, 100 µM, 200 µM, 400 µM, and 800 µM) under standard conditions for KAT I, KAT II, and KAT III (Biochemical Determination Method see Section 2.6), after which the amount of KYNA formed was determined. Six independent experiments were performed.

### 3.2. Effect of Cerebrolysin and D-Cycloserine on KYNA Synthesis in Snail Liver Homogenate

To verify the effects of Cerebrolysin and D-cycloserine on KYNA synthesis, a snail liver homogenate (1:100, *w*/*v*) was incubated in the presence of various concentrations of Cerebrolysin (0.25, 1, 2.5, 5, 10, and 15 µM) or D-cycloserine (50, 100, 200, 400, and 800 µM), under standard assay conditions for KAT I, KAT II, and KAT III (Biochemical Determination Method see Section 2.6). The amount of KYNA formed was then determined. Six independent experiments were performed.

### 3.3. Time-Dependent Formation of KYNA—Effect of L-Kynurenine, Cerebrolysin, and D-Cycloserine

In six independent experiments, the time-dependent synthesis of KYNA was evaluated at incubation times of 1, 2, 3, and 4 h, using two different doses of L-kynurenine (200 µM and 400 µM), Cerebrolysin (0.25 µL and 2.5 µL), and D-cycloserine (200 µM and 400 µM). KYNA was formed in snail liver homogenate (1:100 wt/vol) under standard KAT I, KAT II, and KAT III assay conditions (Biochemical Determination Method see Section 2.6).

## 4. Pharmacological Study—In Vivo

### 4.1. Effect of L-Kynurenine, Cerebrolysin, and D-Cycloserine on KYNA Formation in Ganglia—In Vivo

An in vivo dose–response study was performed in which snails (*Helix pomatia*) were treated with a 100 µL solution containing either L-kynurenine (61.3 mg/mL), Cerebrolysin (176.5 µL/mL), or D-cycloserine (5.1 mg/mL) for one or three consecutive days. The chemicals were carefully administered drop by drop onto the snails’ bodies. After three days, the snails were killed, their ganglia were dissected, and they were frozen at −40 °C until KYNA determination.

### 4.2. Effect of Cerebrolysin and D-Cycloserine on Produced KYNA and Tentacle Lowering, Cycle 1—In Vivo

**Figure 2:**
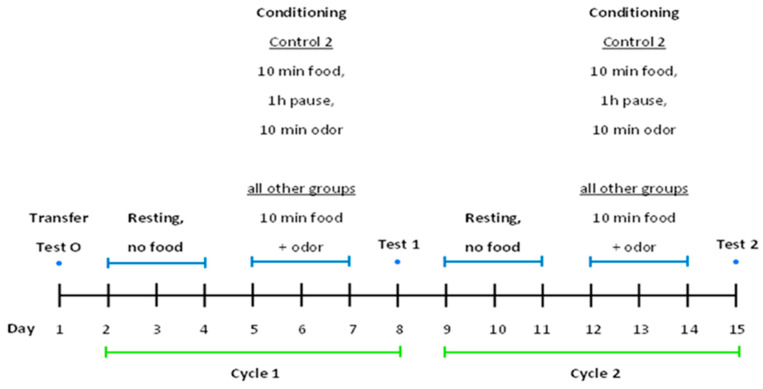
Design of an experiment for pharmacological treatment of *Helix pomatia* snail model for learning and memory. Experiment is described in Section 4.3. Effect of Cerebrolysin and D-Cycloserine on Tentacle Lowering—Cycle 1 and 2–Time Course and Conditioning—In Vivo.

The snails were transferred to individual containers according to their pharmacological treatment group (see Table 1). They were labelled with numbers according to the blend experiment to be performed and food-deprived for four days prior to the start of the experiment (see Figure 2).

The responses of the unconditioned snails were tested on tentacle lowering activity (Test 0, see Figure 2, T0. One hour before conditioning, snails were treated with the solution as indicated in Table 1. Conditioning lasted for the following 3 days, on days 5, 6, and 7. On day 8, the snails’ tentacle activity, response to the dour was tested and recorded T1 (day 8, see Figure 2), and the snails were killed and stored at −40 °C, then dissected ganglia were used for KYNA determination, according to the Material Methods. The behaviors of snails were evaluated.

### 4.3. Effect of Cerebrolysin and D-Cycloserine on Tentacle Lowering—Cycle 1 and 2—Time Course and Conditioning—In Vivo

The snails were treated in a similar way to those in the experiment with Cycle 1, with the addition of the corresponding controls. The responses of the unconditioned snails were tested and recorded 4 days before the start of conditioning (Figure 2 Test 0), (T0). They were then transferred to their individual containers and food-deprived for four days until the start of conditioning. Conditioning lasted for the next 3 days. Conditioning and testing were performed in two cycles (Figure 2: Cycle 1, Test 1 (T1); Cycle 2, Test 2 (T2)). Conditioning took place on days 5, 6, and 7 (three days), as described according to the method before [40,41]. On day 8, the snails’ response to the odor was tested and recorded T1 (see day 8, Figure 2). On days 9, 10, and 11, the snails were allowed to rest in their containers. On days 12, 13, and 14, the snails were conditioned, and on day 15, the response to the odor was again tested and recorded T2 (see Figure 2).

**Figure 3:**
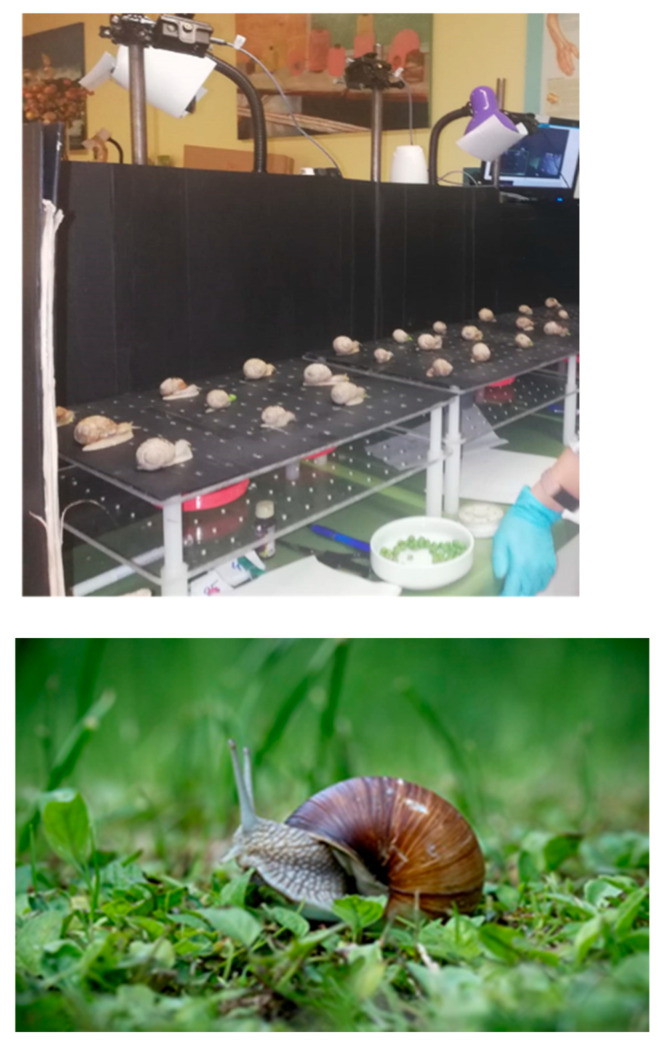
Experimental table for memory test. Snail *Helix pomatia*.

For conditioning, the snails were placed on a test table (Figure 3), a plastic surface with small holes in it and legs at the edges (40 cm × 32 cm × 19.5 cm, length × width × height). Beneath the first plastic surface, at a distance of 6.8 cm, is another surface on which an odor can be placed. The scent used was Air Wick Essential Mist Relaxing Lavender. A few drops of the fragrance oil are dripped onto a piece of paper towel and placed on the second plastic surface. The fragrance oil (odor) represents the conditioned stimulus (CS). A pea is placed in front of each snail, and snails are allowed to eat for 10 min. The pea is the unconditioned stimulus (US).

Snails of control group 1 (CO1) were simultaneously conditioned on a plastic surface (Figure 3). Control group 2 (CO2) receives CS and US separately; i.e., the animals are placed on the plastic surface with a pea in front of each snail and allowed to eat for 10 min, but without the CS (odor). After 1 h, they are exposed to the odor for 10 min.

### 4.4. Testing and Video Analysis

Each snail was placed separately on the same plastic surface (see Figure 3) used for conditioning, with the odor placed on the bottom surface as described for the conditioning experiments. The behaviour of each snail was recorded using a Crosstour Action CT 9000 camera (Electronics B07F3F63SX ZG-CT9000-NEW: 723172909023) (Crosstour, Yuehai Street, Nanshan District, Shenzhen, China), connected to a computer via the ManyCam/Paltalk, Inc. software (version: 9.2.0.4). The Crosstour Action Camera CT 9000 box contains instructions on performing video observation. Each snail’s response to the odor was recorded for 2 min.

A single video contains three windows, one for each camera. The videos were edited using Adobe Premiere Pro to obtain an individual video for each snail. The position of the snails’ tentacles in each video was analyzed using the DeepLabCut software (version: 2.3.11).

All experiments were performed in a blinded manner. The snails were labelled with numbers, and the experiments were carried out. These numbers were then used to form the pharmacological groups.

## 5. Data Analysis

All data are presented as the mean ± standard error. One-way ANOVA and Tukey’s HSD test, or Student’s *t*-test, were used for statistical analysis. Each sample was tested in duplicate. The levels of statistical significance are as follows: * *p* < 0.05, significant compared to the control group; ** *p* < 0.01, significant compared to the control group; *** *p* < 0.001, significant compared to the control group. Microsoft Excel, which is included in Microsoft Office 365 and Microsoft Office LTSC Professional Plus 2024, was used to create the charts.

## 6. Results—In Vitro Study

### 6.1. Dose–Response of L-Kynurenine

**Figure 4:**
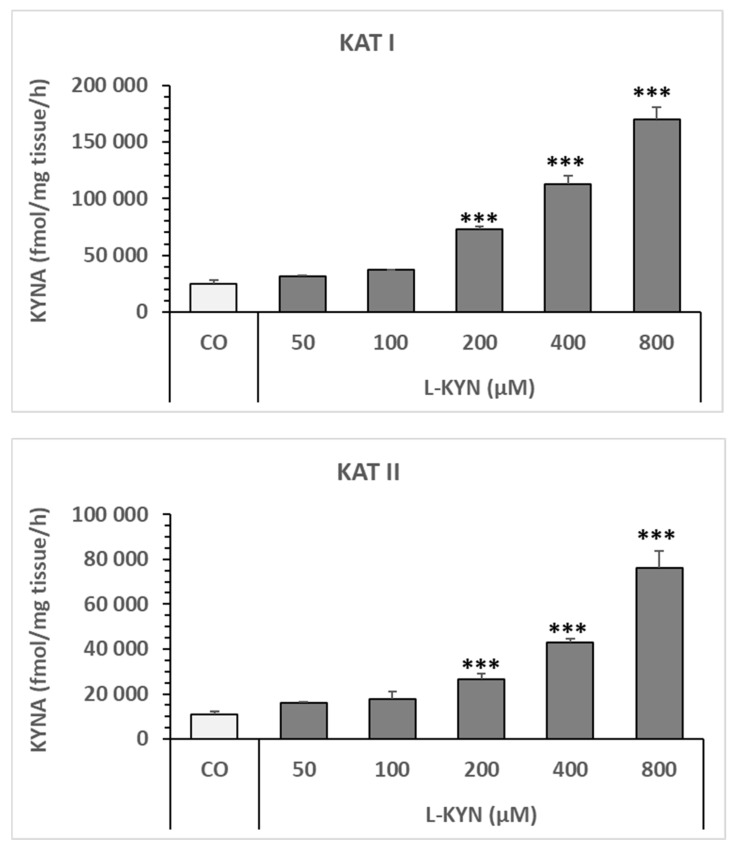

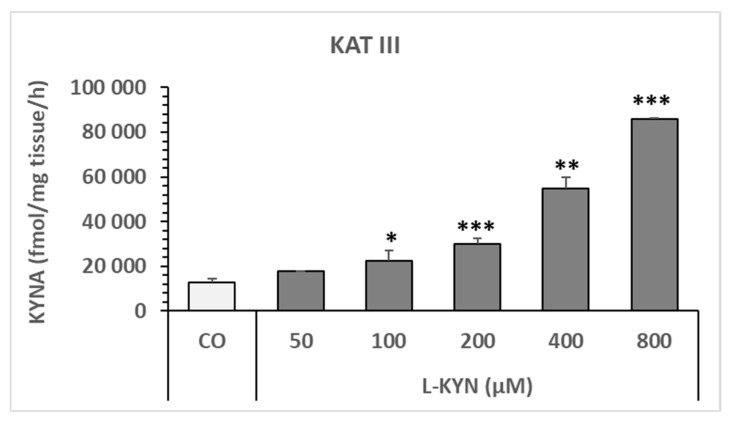
Effect of various doses of L-kynurenine on KYNA formation in *Helix pomatia* snail liver homogenates. KATs activities of the control are 25.02 ± 2.8 (N = 8) for KAT I; 10.87 ± 1.33 (N = 8) for KAT II; 12.75 ± 01.54 (N = 8) for KAT III in pmol/mg wet tissue weight/h. Data expressed in mean ± SEM. Numbers of data used for each concentration is N = 4. Significance of differences: * *p* < 0.05; ** *p* < 0.01; *** *p* < 0.001 vs. the corresponding control. Abbreviation used: control (CO); L-kynurenine (L-KYN). Number of data (N).

KYNA formation increased significantly in a dose-dependent manner in snail liver homogenate at concentrations of 50 µM, 100 µM, 200 µM, 400 µM, and 800 µM of L-kynurenine. The effect on KYNA synthesis was significant in the presence of 200 µM and high doses of L-kynurenine. KAT I was the most effective at forming KYNA, followed by KAT III and KAT II (see Figure 4).

Statistics for KAT I (Figure 4): One-way analysis of variance (ANOVA) revealed significant differences between the control (CO) group and the different L-KYN doses for KAT I: F = 282.2938726, *p* = 1.59482 × 10^−7^. One-way ANOVA between the CO group and each L-KYN dose revealed the following significances: CO vs. 50 µM L-KYN (F = 1.05186486, *p* = 0.335086) and CO vs. 100 µM L-KYN (F = 3.640056383, *p* = 0.092888832123) were not significant; while CO vs. 200 µM L-KYN (F = 111.6820608, *p* = 9.55867 × 10^−7^), CO vs. 400 µM L-KYN (F = 165.242429278, *p* = 1.525777 × 10^−7^), and CO vs. 800 µM L-KYN (F = 282.2938726, *p* = 1.59482 × 10^−7^) were significant.

Statistics for KAT II (Figure 4): A one-way ANOVA revealed significant differences between CO and the various L-KYN doses for KAT II; F = 52.68940622, *p* = 9.4466663 × 10^−10^. A one-way ANOVA of CO and each L-KYN dose revealed the following significances: CO vs. 50 µM L-KYN (F = 2.726821631, *p* = 0.133070219) and CO vs. 100 µM L-KYN (F = 3.414248142, *p* = 0.097697226) were not significant; while CO vs. 200 µM L-KYN (F = 34.33469187, *p* = 0.000109354), CO vs. 400 µM L-KYN (F = 170.901631, *p* = 4.801344 × 10^−8^), and CO vs. 800 µM L-KYN (F = 163.6580978, *p* = 4.45661 × 10^−7^) were statistically significant.

Statistics for KAT III (Figure 4): One-way ANOVA revealed significant differences between CO and different doses of L-KYN for KAT III; F = 45.61672269, *p* = 2.93378 × 10^−9^. One-way ANOVA between CO and each dose of L-KYN revealed the following significances: CO vs. 50 µM L-KYN and CO vs. 100 µM L-KYN were not significant (F = 2.017346562, *p* = 0.189212631; F = 2.017346562, *p* = 0.189212631, respectively); while CO vs. 200 µM L-KYN, CO vs. 400 µM L-KYN, and CO vs. 800 µM L-KYN were significant (F = 27.43334038, *p* = 0.000277609; F = 80.2562474, *p* = 2.19167 × 10^−6^; and F = 404.1630581, *p* = 8.67423 × 10^−9^, respectively).

### 6.2. Effect of Cerebrolysin and D-Cycloserine in Snail Liver Homogenate—In Vitro

**Figure 5:**
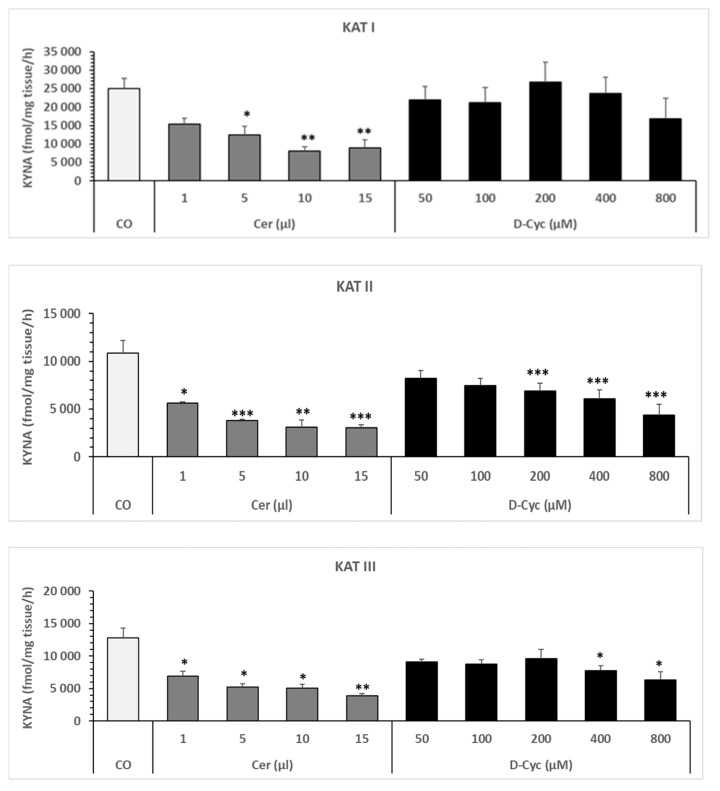
Effect of various doses of Cerebrolysin and D-cycloserine on KYNA formation in *Helix pomatia* snail liver homogenates. KATs activities of the control snail liver homogenate were: 25.02 ± 2.8 (N = 8) for KAT I; 10.87 ± 1.33 (N = 9) for KAT II; and 12.75 ± 01.54 (N = 9) for KAT III, expressed in (pmol/mg wet tissue weight/h). Data represent mean ± SEM. The following doses of Cerebrolysin were used: 1 µL (N = 4); 5 µL (N = 4); 10 µL (N = 4); 15 µL (N = 4). The following doses of D-Cyc were used: 50 µM (N = 4); 100 µM (N = 4); 200 µM (N = 9); 400 µM (N = 9); 800 µM (4). Significance of differences: * *p* < 0.05; ** *p* < 0.01; *** *p* < 0.001 vs. the corresponding control. Abbreviations used: Control (CO); Cerebrolysin (Cer); D-cycloserine (D-Cyc). Numbers of independent measurements are given in parentheses (N).

Cerebrolysin reduced the formation of KYNA via inhibition of KAT I, KAT II, and KAT III activities in snail liver homogenate in a dose-dependent manner (see Figure 5, left column). Boiled Cerebrolysin had no effect on KYNA formation.

D-cycloserine decreased KYNA synthesis in a dose-dependent manner in liver homogenate (Figure 5, right column). The blocking effect on KYNA synthesis was significant in the presence of 200 µM and high doses of D-cycloserine. The inhibitory effect was strongest on KAT II and weaker on KAT III. KAT I was moderately affected by D-cycloserine, though not significantly (Figure 5).

Statistics for KAT I, Cer (Figure 5, left column): A one-way ANOVA revealed significant differences between CO and all doses of Cer for KAT I (Figure 5); F = 7.492689167, *p* = 0.000845609. A one-way ANOVA of KAT I between CO and each dose of Cer revealed the following significances: CO vs. 1 µL was not significant (F = 4.564555071, *p* = 0.058380143); CO vs. 5 µL was significant (F = 7.151487928, *p* = 0.02332272); CO vs. 10 µL was significant (F = 14.77383354, *p* = 0.003245556); and CO vs. 15 µL was significant (F = 11.8844931, *p* = 0.006253477).

Statistic for KAT I, Cer, (Figure 5, left side): A one-way ANOVA analysis revealed significant differences between CO and all doses of Cer for KAT I (Figure 5); F = 7.492689167, *p* = 0.000845609. A one-way ANOVA analysis of KAT I between CO and each dose of Cer revealed the following significances: CO vs. 1 µL was not significant (F = 4.564555071, *p* = 0.058380143); CO vs. 5 µL was significant (F = 7.151487928, *p* = 0.02332272); CO vs. 10 µL was significant (F = 14.77383354, *p* = 0.003245556); and CO vs. 15 µL was significant (F = 11.8844931, *p* = 0.006253477).

Statistics for KAT II, Cer (Figure 5, left column). A one-way ANOVA revealed significant differences between CO and all doses of Cer for KAT II (Figure 5); F = 9.685168643, *p* = 0.00015902. A one-way ANOVA of KAT II between CO and each dose of Cer revealed the following significances: CO vs. 1 µL, F = 5.864217012, *p* = 0.033909768; CO vs. 5 µL, F = 10.67198871, *p* = 0.007507721; CO vs. 10 µL, F = 12.09122266, *p* = 0.00517351; and CO vs. 15 µL, F = 12.8982009, *p* = 0.004233177.

Statistics for KAT III, Cer (Figure 5, left column). A one-way ANOVA revealed significant differences in KAT III (Figure 5) between CO and all doses of Cer; F = 7.955090864, *p* = 0.00052363. A one-way ANOVA of KAT III between CO and each dose of Cer revealed the following significances: CO vs. 1 µL Cer, F = 5.1155001432, *p* = 0.044954025; CO vs. 5 µL Cer, F = 8.684629431, *p* = 0.013281175; CO vs. 10 µL Cer, F = 9.108852527, *p* = 0.011695613; and CO vs. 15 µL Cer, F = 12.2910309, *p* = 0.004919327.

Statistics for KAT I, D-Cyc (Figure 5, right column). A one-way ANOVA for KAT I showed no significant differences between CO and all doses of D-Cyc; F = 0.389097361, *p* = 0.852566898. A one-way ANOVA of KAT I between CO and each dose of D-Cyc revealed the following significant results: CO vs. 50 µM D-Cyc, F = 0.366812598, *p* = 0.55824167; CO vs. 100 µM D-Cyc, F = 0.519830891, *p* = 0.487427006; CO vs. 200 µM D-Cyc, F = 0.072556347, *p* = 0.791320515; CO vs. 400 µM D-Cyc, F = 0.05283496, *p* = 0.821306762; and CO vs. 800 µM D-Cyc, F = 1.805238797, *p* = 0.20877293.

Statistics for KAT II, D-Cyc (Figure 5, right column). A one-way ANOVA for KAT II showed significant differences between CO and all doses of D-Cyc; F = 3.559727969, *p* = 0.011686292. A one-way ANOVA of KAT II between CO and each dose of D-Cyc revealed the following significances: CO vs. 50 µM was not significant (F = 1.38279973, *p* = 0.264447856); CO vs. 100 µM was not significant (F = 2.309110205, *p* = 0.156825344); CO vs. 200 µM D-Cyc was significant (F = 5.329402764, *p* = 0.035630300); CO vs. 400 µM D-Cyc was significant (F = 7.39174044, *p* = 0.015850279); and CO vs. 800 µM D-Cyc was significant (F = 7.904086782, *p* = 0.016925441).

Statistics for KAT III, D-Cyc (Figure 5, right column). A one-way ANOVA for KAT III showed no significant differences between CO and all doses of D-Cyc; F = 2.331065848, *p* = 0.065043161. A one-way ANOVA of KAT III between CO and each dose of D-Cyc revealed the following significant results: CO vs. 50 µM D-Cyc, F = 2.092875248, *p* = 0.175875399; CO vs. 100 µM D-Cyc, F = 2.43317178, *p* = 0.147081902; CO vs. 200 µM D-Cyc, F = 1.952107639, *p* = 0.181436461; CO vs. 400 µM D-Cyc, F = 6.8322953, *p* = 0.01955152; and CO vs. 800 µM D-Cyc, F = 5.791025125, *p* = 0.034832366.

### 6.3. Time Dependence of KYNA Formation—In Vitro Study

**Figure 6:**
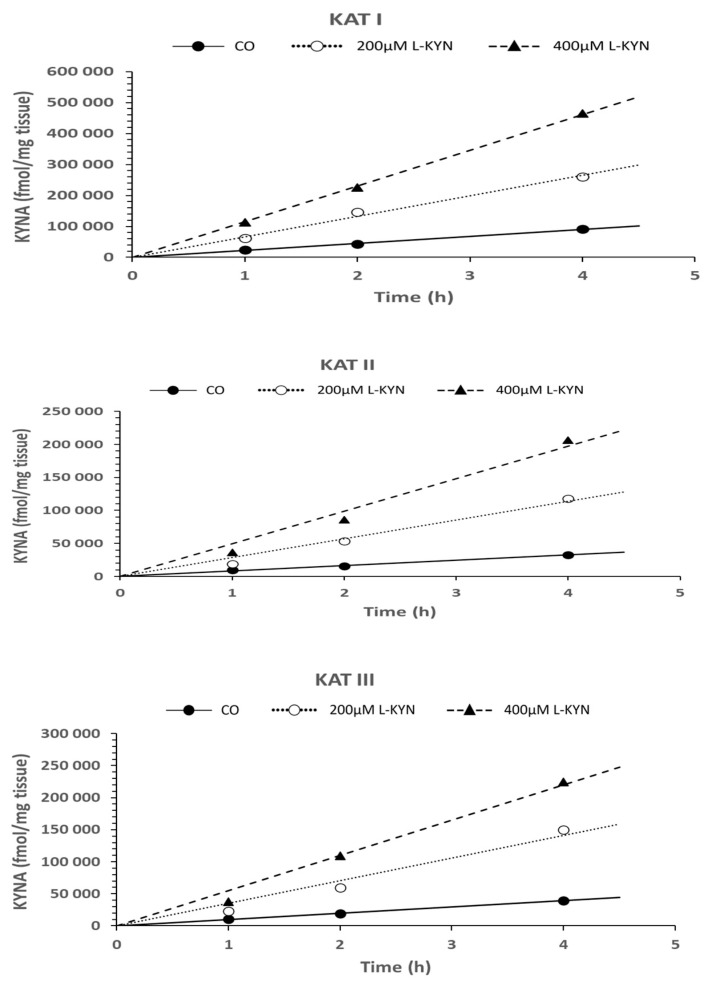
Time dependence of KYNA synthesis using two concentrations of L-kynurenine (L-KYN) (200 µM and 400 µM) in the liver homogenate of *Helix Pomatia* snail for 1 h, 2 h, and 4 h of incubation time using assays for KAT I, KAT II, and KAT III. Data represent mean ± SEM, in (fmol/mg tissue/h). Data numbers independent measurements (N) are given in parentheses: CO 1 h (N = 5), CO 2 h (N = 7), CO 4 h (N = 5); 200 µM L-KYN 1 h (N = 4), 200 µM 2 h (N = 4), 200 µM L-KYN 4 h (N = 4); 400 µM L-KYN 1 h (N = 4), 400 µM L-KYN 2 h (N = 4), 400 µM L-KYN 4 h (N = 4). Abbreviation used: control (CO); L-kynurenine (L-KYN).

Utilizing two concentrations of L-kynurenine (200 µm and 400 µM), the formation of KYNA in snail liver homogenate, using an assay for KAT I, KAT II, and KAT III as described in Materials and Methods, exhibited a time-dependent formation and linear pattern up to 4 h of incubation (see Figure 6).

### 6.4. Time Dependence of KYNA Formation in the Presence of Cerebrolysin—In Vitro

**Figure 7:**
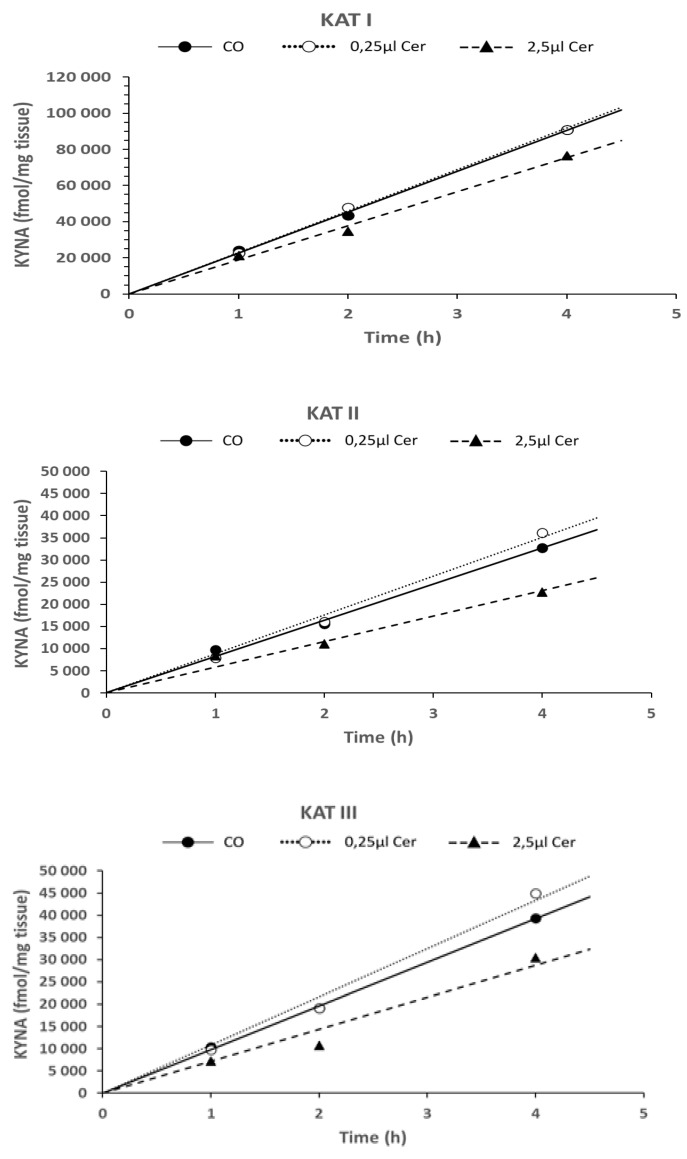
Time dependence of KYNA synthesis in the presence of two concentrations of Cerebrolysin (0.25 µL and 2.5 µL) in the *Helix pomatia* snail liver homogenate for 1 h, 2 h, and 4 h of incubation time under standard assay conditions, as described in Section 2. Data represent mean ± SEM, in (fmol/mg tissue/h); KATs activities are presented in fmol/mg tissue/h. Data numbers (N) are given in parentheses for CO 1 h (N = 5), CO 2 h (N = 7), CO 4 h (N = 5); 0.25 µL Cer 1 h (N = 5), 0.25 µL Cer 2 h (N = 7), 0.25 µL Cer 4 h (N = 5); 2.5 µL Cer 1 h (N = 5), 2.5 µL Cer 2 h (N = 7), 2.5 µL Cer 4 h (N = 5). Abbreviations used: control group (CO); Cerebrolysin (Cer).

The investigation of the effect of two concentrations of Cerebrolysin (0.25 µL and 2.5 µL) demonstrated a time-dependent and linear decrease in KYNA synthesis, extending up to four hours of incubation with KAT I, KAT II, and KAT III, in the presence of a higher dose of Cerebrolysin (2.5 µL), as illustrated in Figure 7, respectively. Using a low dose of Cerebrolysin (0.25 µL), the effect on KYNA synthesis was mostly not present, which was comparable with the control group.

### 6.5. Time Dependence of KYNA Formation in the Presence of D-Cycloserine—In Vitro

**Figure 8:**
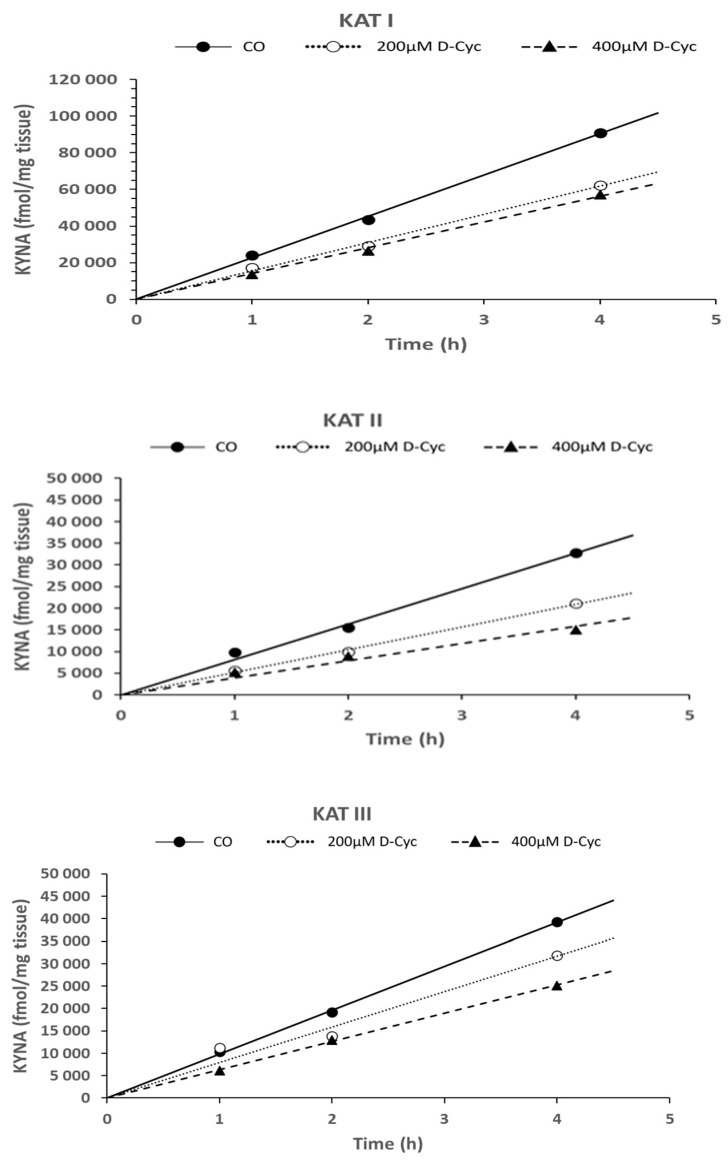
Time dependence of KYNA synthesis in the presence of two concentrations of D-cycloserine (200 µM and 400 µM) in the *Helix pomatia* snail liver homogenate for 1 h, 2 h, and 4 h of incubation time under standard assay conditions, as described in Section 2. Data represent mean ± SEM; KAT activities are presented in fmol/mg tissue/h. Data numbers (N) are given in parentheses: CO 1 h (N = 5), CO 2 h (N = 7), CO 4 h (N = 5); 200 µM D-Cyc 1 h (N = 5), 200 µM D-Cyc 2 h (N = 7), 200 µM D-Cyc 4 h (N = 5); 400 µM D-Cyc 1 h (N = 5), 400 µM 2 h (N = 7), 400 µM 4 h (N = 5). Abbreviation used: control group (CO); D-cycloserine (D-Cyc).

The investigation of the effect of D-cycloserine (200 µM and 400 µM) demonstrated a time-dependent and linear decline in KYNA formation, extending up to 4 h of incubation. This decline was observed in the presence of KAT I, KAT II, and KAT III at 200 µM and 400 µM doses, as depicted in Figure 8, respectively.

Student’s *t*-test statistics for Figure 6, Figure 7 and Figure 8: The Student’s *t*-test significance for KAT I, KAT II, and KAT III activities was calculated. The data corresponding to 1 h and 2 h, 1 h and 4 h, and 2 h and 4 h are displayed in Table 2 (see Table 2a–c).

## 7. Results—Study In Vivo

### 7.1. Effect of Dose–Response of L-Kynurenine, Cerebrolysin, and D-Cycloserine on KYNA Formation

#### 7.1.1. Effect of L-Kynurenine

**Figure 9:**
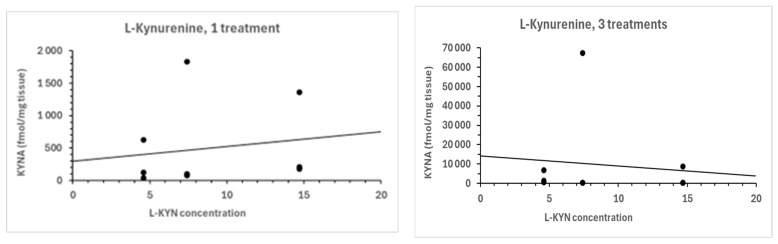

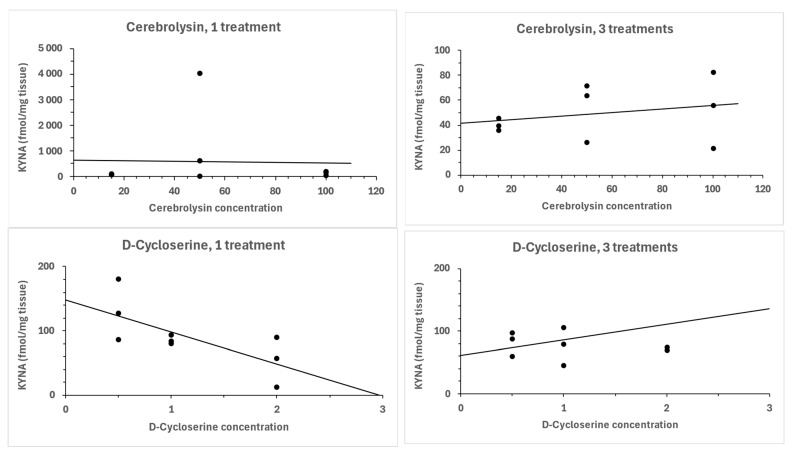
Dose-response of L-kynurenine (4.6 mg, 7.4 mg, and 14.7 mg/10 g of body weight) treatment on KYNA formation in the ganglia of the snail *Helix pomatia* using one or three treatments. Dose-response of Cerebrolysin (15 µL, 50 µL, and 100 µL/10 g of body weight) treatment on KYNA formation in the ganglia of the snail *Helix pomatia* using one or three treatments, in vivo. Dose-response of D-cycloserine (0.5 mg, 1 mg, and 2 mg/10 g of body weight) treatment on KYNA formation in the ganglia of the snail *Helix pomatia* using one or three treatments, in vivo. Data represent mean ± SEM; number of independent data are in parentheses (N = 3). KYNA levels are expressed in fmol/mg tissue.

Treatment with L-kynurenine (4.6, 7.4, or 14.7 mg/15 g body weight ca., respectively) resulted in a dose-dependent increase in KYNA formation in the ganglia after one day or three days of treatment compared to the control group (see Figure 9), respectively.

#### 7.1.2. Effect of Cerebrolysin

The administration of Cerebrolysin (15, 50, and 100/15 g of body weight ca., respectively) to snails resulted in a dose-dependent decrease in KYNA formation in the ganglia after one or three times of treatment, as compared to the control group (see Figure 9), respectively.

#### 7.1.3. Effect of D-Cycloserine

The administration of D-cycloserine to snails resulted in a dose-dependent reduction in KYNA formation in the ganglia. The treatment groups, which received 0.5 mg, 1 mg, or 2 mg of D-cycloserine/10 g of snail body weight ca., respectively, exhibited a significant decrease in KYNA formation compared to the control groups after one treatment and at a lower dose after three days of treatment (see Figure 9), respectively. A higher dose of D-cycloserine induced a moderate increase in KYNA synthesis (see Figure 9).

### 7.2. Pharmacological Treatment of Snail

#### 7.2.1. Changes of KYNA Formation

**Figure 10:**
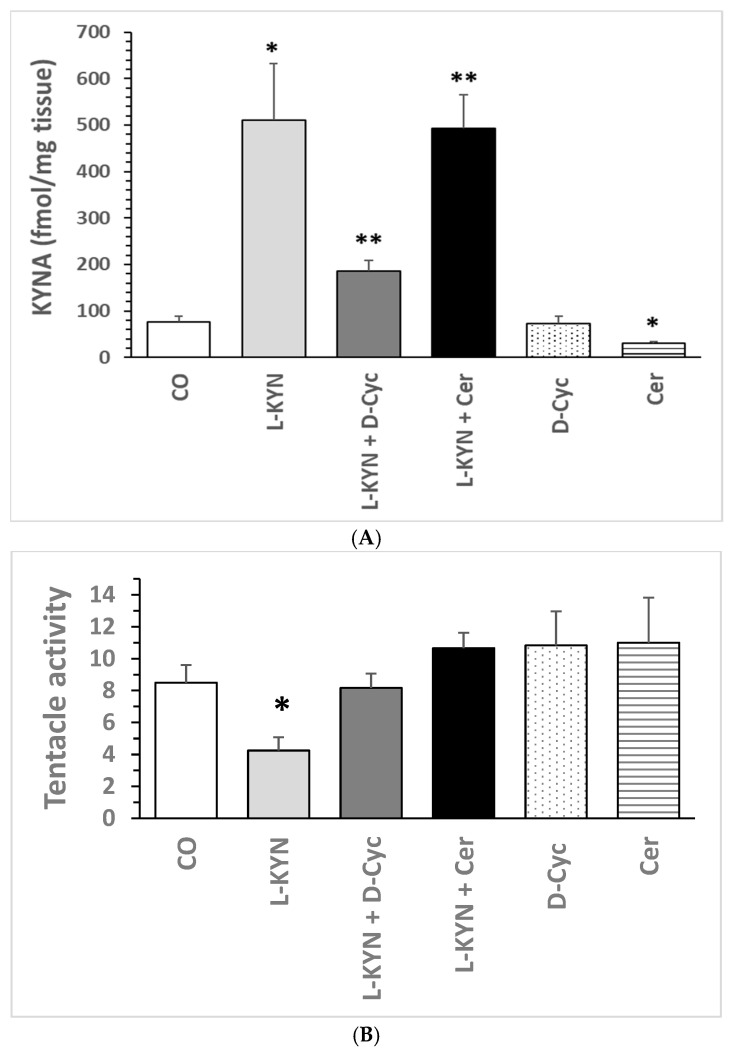
(**A**) Effect of L-kynurenine and anti-dementia drug treatment on KYNA formation in the snail ganglia of *Helix Pomatia*, in vivo. The following concentrations were used: L-kynurenine (0.2 mg/g body weight); Cerebrolysin (1 µL/g body weight); D-cycloserine (0.13 mg/g body weight); mixture of L-kynurenine 0.2 mg/Cerebrolysin 1 µL/g body weight; or mixture of L-kynurenine 0.2 mg/D-cycloserine 0.13/g body weight. After the first cycle, the measurement of tentacle lowering at time T1 was performed, and KYNA levels in ganglia homogenate were determined according to Section 2. Data represent mean ± SEM. Significance of differences: * *p* < 0.05; ** *p* < 0.01; vs. the corresponding control (T0). KYNA levels are presented in fmol/mg tissue. Abbreviations: control (CO); L-kynurenine (L-KYN); D-cycloserine (D-Cyc); Cerebrolysin (Cer). Data numbers are given in parentheses: CO (N = 6); L-Kyn (N = 8); L-Kyn/D-Cyc (N = 6); L-Kyn/Cer (N = 6); D-Cyc (N = 6); Cer (N = 6). (**B**) Effect of L-kynurenine and anti-dementia drug treatment on the tentacle lowering of snail *Helix Pomatia*. The following concentrations were used: L-kynurenine (0.2 mg/g body weight); Cerebrolysin (1 µL/g body weight); D-cycloserine (0.13 mg/g body weight); mixture of L-kynurenine 0.2 mg/Cerebrolysin 1 µL/g body weight; or mixture of L-kynurenine 0.2 mg/D-cycloserine 0.13/g body weight. Behavior is expressed with a number of lowering tentacles. After the first cycle, the measurement of tentacle lowering was performed at time T1. Data represent mean ± SEM. Significance of differences: * *p* < 0.05; vs. the corresponding control (T0). Data numbers are given in parentheses: CO (N = 6); L-Kyn (N = 8); L-Kyn/D-Cyc (N = 6); L-Kyn/Cer (N = 6); D-Cyc (N = 6); Cer (N = 6). Abbreviations: Control (CO); L-kynurenine (L-KYN); D-cycloserine (D-Cyc); Cerebrolysin (Cer).

Treatment of the *Helix pomatia* with L-kynurenine resulted in a substantial increase in KYNA levels within the ganglia (Figure 10A). The treatment with L-kynurenine/D-cycloserine lowered KYNA formation. The co-administration of the L-kynurenine/Cerebrolysin exhibited positive and negative impact on KYNA levels. D-cycloserine or Cerebrolysin exhibited a reduction in KYNA formation compared to the control.

A one-way ANOVA for KYNA levels (Figure 10A) revealed significant differences between all pharmacological groups: F = 8.76677; *p* = 2.55021 × 10^−5^. One-way ANOVA for KYNA levels revealed a significant increase in KYNA concentrations, with a statistical significance of F = 8.29534 and *p* = 0.01383, between CO and L-KYN. Additionally, a trend towards statistical significance was observed in the L-KYN and L-KYN/D-Cyc mixture, with a statistical significance of F = 4.56984 and *p* = 0.055381. However, no statistical significance was detected between the L-KYN and L-KYN/Cer mixtures, with a statistical significance of F = 0.01212 and *p* = 0.91414. Furthermore, a significant difference in KYNA concentrations was observed between the L-KYN/D-Cyc mixture and D-Cyc, with a statistical significance of F = 13.07669 and *p* = 0.00472. Finally, a significant difference in KYNA concentrations was detected between the L-KYN/Cer mixture and Cer, with a statistical significance of F = 0.3464368 and *p =* 0.0001539. A statistically significant relationship was identified between L-KYN/Cer and L-KYN/D-Cyc, with a *p*-value of 0.00394 and a confidence interval of 13.87382. In contrast, no significant variation in KYNA levels was observed between CO and D-Cyc. The variation in KYNA levels in the L-KYN/Cer group could be due to low and high KYNA levels among samples. Storage of the samples could affect the data. This will be explored in the future.

#### 7.2.2. Alteration of Tentacle Lowering

A modification in the degree of activity exhibited by the tentacles has been observed (see Figure 10B). Snail activities were evaluated prior to conditioning (T0) and following cycle test I. L-kynurenine treatment led to a substantial reduction in tentacle lowering compared to the control group (see Figure 10B). The co-administration of L-kynurenine with D-cycloserine or Cerebrolysin effectively negated the effect of L-kynurenine-induced tentacle lowering, resulting in values that were comparable to those observed in the control group. The administration of D-cycloserine or Cerebrolysin, either individually or in combination, resulted in a moderate augmentation of tentacle activity when compared to the control group.

A one-way ANOVA for tentacle-lowering levels (see Figure 10B) between all pharmacological groups demonstrated significant differences; F = 2.59588; *p* = 0.04443. A one-way ANOVA was conducted to assess the statistical significance of the observed reduction in tentacle lowering. The analysis revealed a substantial decrease in tentacle lowering between CO and L-KYN, with statistical significance indicated by an F value of 8.54187 and a *p*-value of 0.01278. Additionally, a significant effect of D-Cyc was observed in the comparison between L-KYN and the mixture of L-KYN and D-Cyc, with statistical significance indicated by an F value of 8.72548 and a *p*-value of 0.01206. However, no significant differences in tentacle lowering were observed between L-KYN and L-KYN/Cer, with statistical significance indicated by an F value of 3.01609 and a *p*-value of 0.11309. A comparison of the effects of L-KYN/D-Cyc and D-Cyc on tentacle lowering revealed no significant differences (F = 0.01044, *p* = 0.92064). Similarly, no significant differences were observed in the changes in tentacle lowering when comparing L-KYN/Cer and Cer (F = 1.11304, *p* = 0.31624). Additionally, no significant differences in tentacle lowering were detected when CO was compared to D-Cyc (F = 0.7916, *p* = 0.039452). Likewise, no significant differences in tentacle lowering were observed when CO was compared to Cer (F = 0.56904, *p* = 0.46803). Finally, a lack of significant differences between D-Cyc and Cer in terms of tentacle lowering was revealed (F = 0.00186, *p* = 0.96648).

## 8. Effect of Pharmacological Treatment on *Helix pomatia*—Behavior of Tentacle Lowering

**Figure 11:**
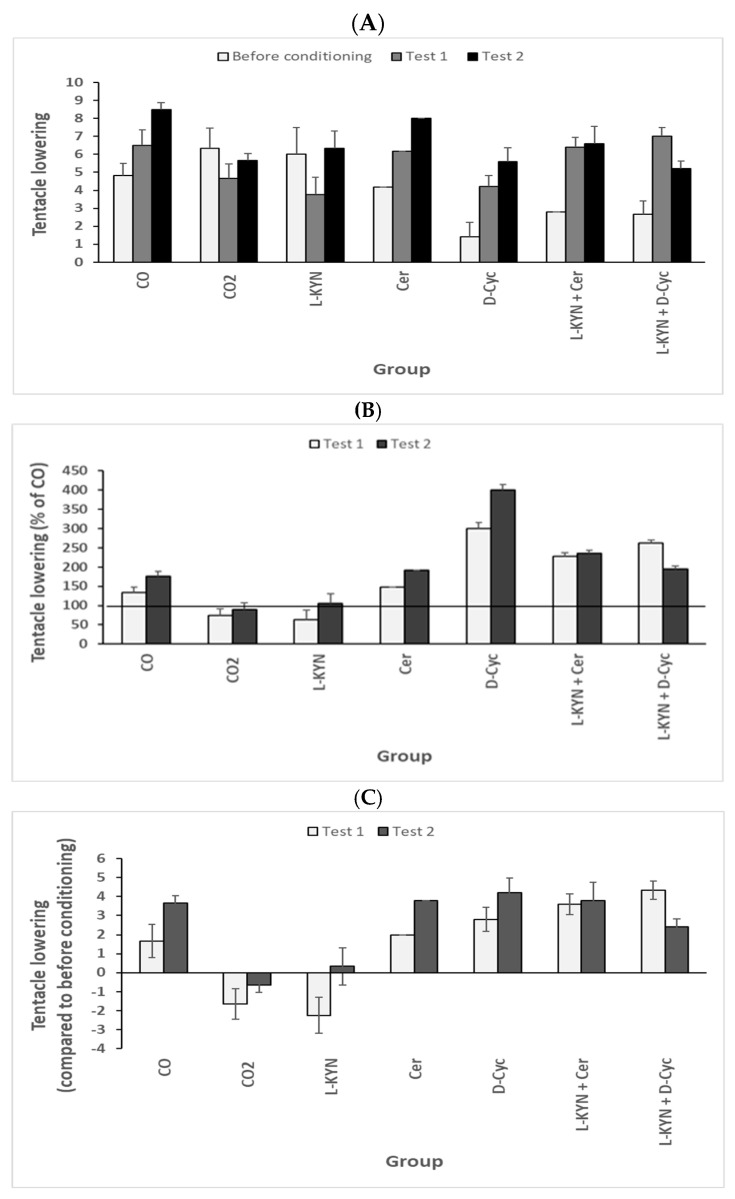
Effect of pharmacological treatment of the snail *Helix pomatia.* Evaluation of the effect of L-kynurenine and pharmacological treatment on tentacle lowering. Used drugs: L-kynurenine (6.13 mg/15 g body weight); Cerebrolysin (17.65 µL/15 g body weight); D-cycloserine (0.51 mg/15 g body weight); mixture of L-kynurenine (6.13 mg)/Cerebrolysin (17.65 µL)/15 g body weight; or mixture of L-kynurenine 6.13 mg/D-cycloserine (0.51 mg)/15 g body weight. (**A**) shows number of tentacle depressions before conditioning; (**B**) shows percentage of CO, horizontal line indicates 100%; (**C**) shows the change in the number of tentacle depressions after conditioning (i.e., the number of tentacle depressions after conditioning minus the number of tentacle depressions before conditioning). Data numbers are given in parentheses: CO (N = 6); CO2 (N = 6); L-Kyn (N = 4); Cer (N = 6); D-Cyc (N = 5); L-Kyn/Cer (N = 5); L-Kyn/D-Cyc (N = 6). Abbreviation: control (CO); L-kynurenine (L-KYN); D-cycloserine (D-Cyc); Cerebrolysin (Cer). Abbreviations were used in the ANOVA description.

In the following experiment, snail activities were tested before the conditioning (T0) and after the first (Test 1) and second (Test 2) test cycles. Data are presented in Figure 11 (Figure 11A–C). In comparing CO with CO2, we observed increased snail tentacle activity due to the presence of odor, indicating a learning process. This learning process was not observed after L-kynurenine treatment (i.e., an additional “stimulus”). We observed the inhibition of tentacle lowering due to L-kynurenine. The treatment with Cerebrolysin and D-cycloserine increased tentacle lowering, indicating increased learning activity. The co-application of mixtures L-kynurenine/D-cycloserine or L-kynurenine/Cerebrolysin abolished the effect of L-kynurenine (see Figure 11, Test 1). In Test 2, a positive effect was observed in all groups except CO2 and L-KYN.

Three different plots indicate the same results: Figure 11A represents the number of tentacle depressions before conditioning; Figure 11B represents this as a percentage of CO; and Figure 11C represents this as the change in the number of tentacle depressions after conditioning (i.e., the number of tentacle depressions after conditioning minus the number of tentacle depressions before conditioning).

One-way ANOVA for tentacle lowering levels in the control group with conditioning (Figure 11, CO) between T0, T1, and T2 measurements shows significant differences (F = 4.67825; *p* = 0.0278). In terms of significance, T0 vs. T1 was most significant, F = 4.95097, *p* = 0.05312; T1 vs. T2 was not significant, F = 0.2063, *p* = 0.66044; T0 vs. T2 was significant, F = 12.47423, *p* = 0.00543. One-way ANOVA for tentacle lowering levels in the Control group without conditioning (Figure 11, CO2) between T0, T1, and T2 measurements revealed no significant differences, F = 1.17284; *p* = 0.33632. In terms of significance, T0 vs. T1 was not significant, F = 1, *p* = 0.21818; T1 vs. T2 was significant, F = 5.82759, *p* = 0.04224; T0 vs. T2 was not significant, F = 0.32558, *p* = 0.58611.

One-way ANOVA for tentacle lowering in the L-KYN group (Figure 11, L-KYN) between T0, T1, and T2 measurements revealed no significant differences, F = 2.70565; *p* = 0.11076. In terms of significance, T0 vs. T1 was not significant, F = 1.83007, *p* = 0.21818; T1 vs. T2 was significant, F = 5.82759, *p* = 0.04224; T0 vs. T2 was not significant, F = 0.32558, *p* = 0.58611. One-way ANOVA for tentacle lowering in the Cer group (Figure 11, Cer) between T0, T1, and T2 measurements revealed significant differences, F = 6.46565; *p* = 0.01026. In terms of significance, T0 vs. T1 was not significant, F = 4.04494, *p* = 0.07203; T1 vs. T2 was not significant, F = 3.32215, *p* = 0.10167; T0 vs. T2 was significant, F = 10.98523, *p* = 0.00902.

One-way ANOVA for tentacle lowering in the D-Cyc group (Figure 11, D-Cyc) between T0, T1, and T2 measurements revealed significant differences, F = 3.7; *p* = 0.04941. In terms of significance, T0 vs. T1 was not significant, F = 1.94595, *p* = 0.19323; T1 vs. T2 was not significant, F = 1.16046, *p* = 0.30667; T0 vs. T2 was significant, F = 12.18232, *p* = 0.00587. One-way ANOVA for tentacle lowering in the L-KYN/Cer group (Figure 11, L-KYN/Cer) between T0, T1, and T2 measurements revealed significant differences, F = 5.02786; *p* = 0.02261. In terms of significance, T0 vs. T1 was significant, F = 6.53768, *p* = 0.03084; T1 vs. T2 was not significant, F = 0, *p* = 1; T0 vs. T2 was significant, F = 6.77922, *p* = 0.02856.

One-way ANOVA for tentacle lowering in the L-KYN/D-Cyc group (Figure 11, L-KYN/D-Cyc) between T0, T1, and T2 measurements revealed significant differences, F = 8.61969; *p* = 0.00363. In terms of significance, T0 vs. T1 was significant, F = 26.40625, *p* = 4.38589 × 10^−4^; T1 vs. T2 was not significant, F = 2.42461, *p* = 0.15387; T0 vs. T2 was not significant, F = 4.13096, *p* = 0.07264.

## 9. Discussion

As demonstrated in the previous study by Kronsteiner in 2023 [43], KYNA formation in *Helix pomatia* brain or liver homogenates was significant and dependent on the L-kynurenine dose. Our research reveals a novel finding: anti-dementia drugs, including Cerebrolysin and D-cycloserine, effectively reduce KYNA synthesis in vitro in *Helix pomatia* liver homogenate. A similar effect was observed in vivo, where brain KYNA levels increased significantly following L-kynurenine treatment. Furthermore, following treatment with Cerebrolysin or D-cycloserine, a significant reduction in KYNA levels was observed in snail ganglia, indicating the blockade of KYNA-synthesizing enzymes (KATs) in the CNS. The effect on the liver homogenate of snails was visible at concentrations as low as 1 µL of Cerebrolysin. This is comparable to the effects observed in rat liver and brain homogenates, as well as in postmortem human brain tissue [31].

The blocking effect of D-cycloserine in snail liver homogenate was observed at 200 µM for KAT II and at 400 µM for KAT III. High doses of D-cycloserine (800 µM) effectively blocked KAT II and KAT III, with the blockade capacity occurring in the following order: KAT II > KAT III > KAT I.

Our pharmacological studies have shown that treating the *Helix pomatia* snail with L-kynurenine (90 nM) increases KYNA levels in its ganglia by around 630%. Furthermore, under these conditions, we observed a significant reduction in tentacle lowering. However, the outcome was altered when L-kynurenine was applied alongside D-cycloserine (1 µM). KYNA levels decreased from 630% to approximately 200% of control levels, and tentacle lowering increased from around 4 to 8. These data suggest that an increase in KYNA of over 300% of control levels can lead to impairment. This correlation has not been presented before, at least not for *Helix pomatia* snails. Data obtained from human material show a strong correlation, indicating high levels of KYNA in patients with memory impairment [25]. These data allow us to speculate that influenza may also cause such transient alterations.

A weak yet significant effect of D-cycloserine (approximately 20% blockade) to block KATs was observed in the livers and brains of rats, as well as in postmortem human brains, at a low concentration of 0.673 µM [32]. Higher levels of D-cycloserine, approximately one hundred times greater, caused a reduction of about 50%. Notably, 673 µM doses of D-cycloserine completely blocked KYNA formation [32]. Interestingly, a similar pattern of KAT inhibition (KAT II > KAT III > KAT I) was revealed in the snail liver homogenate, regardless of whether the D-cycloserine dose was used. This suggests that among KATs, KAT II is particularly sensitive to D-cycloserine treatment regardless of species (snail, rat, or human) and that it may play a pivotal role in various biochemical cellular events depending on the dosage. Indeed, long-term D-cycloserine treatment has caused significant side effects in patients with schizophrenia [48], which may be related to KAT II’s inability to produce more KYNA. The inhibitory effect has also been observed in other species, including rats and humans, in the periphery and/or CNS, at least in vitro [31,32]. Interestingly, we have observed the blocking effect of D-cycloserine and Cerebrolysin in human saliva, too, and the effect was stronger for KAT II, as well [49]. The presence of KAT I, KAT II, and KAT III in human saliva indicates significant involvement in the human body. The function and role of KAT saliva are under investigation. These findings suggest that the pharmacological effects of anti-dementia drugs on L-kynurenine metabolism are similar between species. The effective dose of D-cycloserine to inhibit KAT II activity in vivo is predominant and, therefore, pharmacological management should be implemented.

Snails have previously been proposed [50] and are still used as a model organism for studying learning and memory [39,41,51]. In light of these findings and accumulated biochemical data regarding KYNA synthesis [43], the *Helix pomatia* snail model was used as a “learning and memory model” to evaluate the impact of various pharmacological interventions on the L-kynurenine metabolism process and learning/memory. We found that pre-treating the snails with L-kynurenine increased KYNA levels in the liver and ganglia (up to 630% ca.), and unfortunately, impaired learning ability.

Acebes et al. described the tentacle lowering in the *Helix aspersa* snail as an indicator of learning, memory, attention, and alertness [39,40,41]. Tentacle lowering served as an operational definition of the cognitive domain being measured [50,51], and we have applied it to the *Helix pomatia* snail. We believe that this behavioral event is specific to memory rather than to general arousal or motor suppression. The pharmacological approach using L-kynurenine and the anti-dementia drugs D-cycloserine and Cerebrolysin supports this idea. Tentacle activity decreased following treatment with L-kynurenine, as evidenced by a decline in tentacle lowering. This may indicate impaired learning and memory capacity in the presence of elevated KYNA levels. Similar biochemical and physiological events have been observed in young rats treated with 30 mg/kg of L-kynurenine, in which an increase in KYNA levels, likely in both parts CNS and periphery, led to dramatic memory impairment [2,3].

There are more significant findings related to an enhancement of KYNA levels and the occurrence of cognition and memory impairment in mammals. Connick et al. (1989) demonstrated an increase in KYNA levels in the motor cortex of human subjects with Huntington’s disease [52]. These subjects experienced dementia, though not in the form of Alzheimer’s disease [53]. A significant increase in KYNA levels in the brain after neonatal asphyxia would also indicate subsequent dementia development [54]. Markedly elevated KYNA levels observed in the Kainic acid model of epilepsy [55] support the idea of the development of memory impairment in epileptic patients or in other pathological conditions, such as Down’s syndrome or Hydrocephalus. These conditions have been confirmed to impair cognition and memory, as described in the review [25]. Even in epileptic patients taking the anticonvulsant drug like diazepam, which affects KYNA formation in the brain tissue, impairment of learning and memory has been reported [56,57,58].

Thus, the present study demonstrated for the first time that administering L-kynurenine alongside an anti-dementia pharmaceutical agent resulted in notable behavioral alterations in *Helix pomatia* snails. These alterations were characterized by a reduction in the frequency of tentacle lowering following L-kynurenine treatment alone, and a significant increase in tentacle lowering following the application of L-kynurenine/D-cycloserine or L-kynurenine/Cerebrolysin. These findings support the idea that the positive effects of drugs such as Cerebrolysin and D-cycloserine on learning and memory can be tested using the *Helix pomatia* snail learning and memory model.

Some authors might argue that the mechanistic properties of Cerebrolysin are difficult to interpret due to its complex peptide and amino acid composition. However, we consume complex food mixtures in real life, and our bodies filter these to utilize specific molecules for memory formation. Cerebrolysin is commonly used in clinics, with patients reporting no side effects and feeling well [59,60]. Nevertheless, the company does not recommend this drug for patients with epilepsy (in the instructions of the drug for medical doctors), due to the possible induction of seizures.

Interestingly, Cerebrolysin has been found to reduce microglia activation in vivo [61]; however, not all patients treated with it had decreased KYNA levels, as observed by Kepplinger. It is also important to mention that piglet brains are used for the production of Cerebrolysin. Similarly, we found that KYNA synthesis in the presence of piglet brain tissue (homogenate) is decreased significantly [33].

Furthermore, we have shown that not only piglet brain but also herbs impede KAT activity [34,35]. Furthermore, the formation of KYNA and ANA has been shown to be reduced by many well-known naturally occurring plants, such as sage, lemon balm, and hawthorn berry extract, in vitro [34,35]. Interestingly, hawthorn berry extract has also been found to have anti-dementia properties and to support heart function [62]. In Europe, Crataegutt, a hawthorn berry extract, is produced by Schwabe Austria GmbH in Vienna, and is available from pharmacies as a dietary supplement designed to support the hearts and brains of older adults.

A previous in vitro study demonstrated that a high D-cycloserine dose (≥673 µM, in vitro) blocks KYNA formation in human homogenate completely [32]. The present study also demonstrated a significant reduction in KYNA formation by D-cycloserine between 500 µM and 800 µM. However, in vivo studies revealed that higher D-cycloserine doses (>2 mg) were not accompanied by a reduction in KYNA levels but rather by a notable moderate increase. Our observations revealed that KAT I activity remained unchanged in the presence of a high D-cycloserine dose. Conversely, KYNA formation was observed at a level comparable to that observed in the Control group, with a notable increase in KYNA synthesis also observed in some cases.

A study on KAT I in humans revealed moderate reductions in KAT I, and even increases were observed in some cases [32,34]. Goff’s research suggests that administering D-cycloserine to patients diagnosed with schizophrenia has a positive effect [48]. However, long-term use leads to deterioration [48]. This observation potentially explains why higher D-cycloserine is less effective at reducing KYNA levels, and increases ‘tentacle lowering’ over extended treatment periods (Baran’s observation). Notably, Goff (2017) found that reducing the dosage enhanced the efficacy of D-cycloserine treatment in patients diagnosed with schizophrenia [63]. Goff’s observations are in line with the D-cycloserine dose on KYNA levels in ganglia, where the KYNA levels were comparable to those of the Control, or even moderately increased.

It is important to note that another L-kynurenine metabolite, anthranilic acid (ANA) (see Figure 1), is also elevated in individuals with schizophrenia [64]. A similar pattern of increased ANA and KYNA (ANA >> KYNA) has been observed in stroke patients, as well [65]. Both patient groups have memory impairment. Besides the drug therapy, the Zeptoring treatment, called Stochastic Resonance Therapy, was effective for both patient groups [25]. All patients reported feeling well, likely due to the reduction of KYNA and, to a lesser extent, ANA observed in healthy subjects [66].

Previously, we reported on the significant, dose-dependent effects of L-kynurenine and its metabolites, including KYNA, xanthurenic acid (XAN), and ANA, on respiratory parameters in the mitochondria of rat brains, hearts, and livers [67].

A study on the influence of aging on KATs in rat brain mitochondria revealed an increase in the brain and in the heart homogenates between 3 and 26 months; however, in the mitochondrial suspensions of brain and heart, the KAT II and KAT III activities were only moderately higher [68]. No alteration of KAT I activities was found in the homogenates and mitochondrial suspensions prepared from brain and hearts of 3-, 12-, and 26-month-old animals [68]. The revealed data indicate that under physiological condition the age-dependent increase in KYNA levels in the brain is due to an involvement of an age-dependent increase in KAT II and KAT III activities. Alterations in KAT I activity under physiological and/or pathological conditions are probably not related to the aging process, but rather depend on the cellular environment, at least in rats. The revealed data indicate the complexity of KYNA formation and the difficulty of KATs’ role.

Because differences in the effects of tryptophan metabolites on mitochondrial respiratory parameters have been observed in the heart, brain, and liver [67], we believe the anti-dementia drug may impact not only KYNA but also XAN and, notably, ANA. These data suggest that brain mitochondria are impaired in the presence of increased XAN levels, and that the application of anti-dementia drugs might correct these levels and the accompanying pathological events, as we recently suggested [25].

No effect of millimolar quinolinic acid doses on mitochondrial respiratory parameters in the heart, liver, or brain of rats was detected [67]. Ageing: the quality of the mitochondrial respiratory parameters in the brain, heart, and liver was unaffected by ageing, at least in healthy rats [68]. Some publications have suggested that quinolinic acid plays a more significant role in the development of dementia [69]. Previous studies have shown that high doses of L-kynurenine metabolites, such as KYNA, 3-hydroxykynurenine, and 3-hydroxyanthranilic acid, significantly affect rat mitochondrial respiratory parameters. However, these effects were not observed in the presence of quinolinic acid [67]. Furthermore, our study suggests that applying KAT inhibitors may increase quinolinic acid synthesis (see Figure 1). Contrary to expectations, tentacle activity increased rather than decreased in vivo study.

The neurodegenerative effects of quinolinic acid are well-documented in HIV-1-infected patients and may significantly impact memory and cognition [70]. These quinolinic acid-induced biochemical events are related to cell loss and the proliferation of cells that synthesize KYNA following neurodegeneration [13,71,72]. Notably, KAT I activity increased significantly under various pathological conditions and after HIV-1 infection [73]. Despite KYNAs’ neuroprotective and anticonvulsant properties [20,21], an enhancement of KYNA cannot stop or delay neurodegenerative events. Although many scientists believe that the enhancement of KYNA lowers the effects. A marked increase in KYNA levels has been observed in the Kainic acid rat epilepsy model, in rats without and in rats with generalized seizures [55]. Is KYNA involved in the propagation of seizures? D-cycloserine (160 mg/kg) shows anticonvulsive activities in the Kainic acid rat epilepsy model [74], or in electrically induced tonic seizures in mice [75].

Which KYNA dose is the most valuable? I would argue that increasing KYNA doses by 300% poses a significant health risk and could lead to memory impairment. Lower doses of D-cycloserine (1 µM) are effective in blocking KYNA formation. However, both the highest and lowest KYNA levels can be fatal. Anticonvulsant and neuroprotective doses of KYNA are physiological concentrations. This is the KYNA phenomenon.

Notably, our data suggest that the ability of drugs to increase and/or decrease KYNA synthesis is a significant indicator of their potential as anti-dementia drugs. Which KAT might be more effective for anti-dementia drugs in vivo? Is questionable. This needs further research. Interestingly, increased water consumption was found to reduce KYNA levels in snails, as observed by Kronsteiner. This finding correlates with the slight memory impairment experienced by individuals who reduced their daily liquid intake, including water. Doctors recommend controlling water consumption to improve mood, memory, and health, particularly in older adults. Modulating KYNA synthesis may also significantly impact processes responsible for energy supply.

Changes in behavior are easy to detect by observing the rats themselves, but what about using cameras to assess quality? I wrote a report on the behavior of rats in the Kainic acid model using a rating scale [55,74], and I found working with the rating scale very helpful. Videos can limit expression and miss small, mostly undetectable events that are physiologically important. Therefore, I recommend trying both methods in the snail model of learning and memory. One disadvantage is the nature of the snail; they are very slow, and humans want to see immediate results.

According to Owens et al. (2017) and other scientists, biological learning processes originate in neurons, which are electrically activated brain cells [76]. Learning occurs through changes in the strength and number of neural pathways, a process known as synaptic plasticity [76]. Reducing endogenous KYNA formation enhances extracellular glutamate, neuronal activity, and hippocampal plasticity [37]. Therefore, synaptic plasticity increases when a motor task is repeated, and repeated activities are crucial for behavioral learning and memory formation. A pharmacological approach used in the study was helpful to better understand the biochemical processes involved in tentacle lowering and its role in memory formation.

In addition to neurons, which are highly specialized for transmitting electrical signals rapidly, some glial cell properties are well-suited for participating in complex cognitive functions. Incorporating glia into the mechanisms by which the nervous system functions may help answer long-standing questions about the cellular mechanisms of learning and cognition, as suggested by Fields et al. in 2014 and 2015 [77,78].

The genetic factor influencing the biochemistry of tryptophan metabolism in different species may also play a pivotal role. Additionally, biochemical metabolism differs significantly among species as they age, supporting the importance of low KYNA levels [25]. These data suggest that a slow metabolism of L-kynurenine with age is advantageous for maintaining a good memory.

KYNA also demonstrates an interesting action, acting as an agonist of the orphan G protein-coupled receptor GPR-35 [18,19]. Regarding the importance of the involvement of KYNA and XAN in relation to the GPR-35 receptor in the gut microbiota, specific GPR-35-positive signals have been detected in the gastrointestinal tract [18,19]. Lowering KYNA levels in ageing birds reveals remarkable biochemical conditions [25]. Also, ANA acts as an agonist of the orphan G protein-coupled receptor GPR109A, which may play a pivotal role [79]. In this excellent and clear work published in 2024 by Oxenkrug, I found notable data supporting the importance of ANA [79]. Similarly, my co-worker Kepplinger observed significant involvement of the ANA/KYNA ratio in the blood of stroke patients following repetitive transcranial magnetic stimulation (rTMS) treatment [65]. The ANA/KYNA ratio increased moderately and significantly after the third stimulation. Alteration of the ANA/KYNA ratio following rTMS was significant with respect to clinical improvement in patients, including improvements in cognition and memory [65].

An interesting paper by Berlinguer-Palmini et al. (2013) demonstrated that the activation of GPR35 reduces Ca^2+^ transients and contributes to the KYNA-dependent reduction in synaptic activity at the CA3-CA1 synapses in the hippocampus [80], indicating the presence of GPR35 receptors in the brain. A further notable observation has been made by Deng et al. (2025) [81], whose work explores the interaction between the gut microbiota and the cyclic adenosine monophosphate–protein kinase A signaling pathway, suggesting a potential therapeutic approach for neurodegenerative diseases. Furthermore, regarding this interaction, KYNA may modulate the relationship between AMP and ATP in the hippocampus, which could influence memory capacity [80,82]. This is an intriguing and intricate mechanism involving numerous variables that fluctuate based on energy supply and biochemical processes.

## 10. Conclusions

Regarding the role of KYNA in learning memory impairment, our data strongly suggest that increased KYNA formation is involved. An anti-dementia drug blocks the formation of KYNA, reduces its levels, and recovers learning ability. The *Helix pomatia* snail memory model, probably, is a valuable tool for evaluating the effects of certain compounds on memory and the biochemistry of the kynurenine pathway in vivo. Within this model, using pharmaceuticals or natural agents that can modulate kynurenine metabolic activity in the central and peripheral nervous systems by reducing KYNA levels through KAT inhibition represents a promising approach to alleviating or preventing acute cardiovascular and neurological exacerbations in patients with dementia and/or inflammation. Additionally, this approach could be used prophylactically to promote cognitive health throughout the human lifespan.

In the future, we would like to test the biochemical activity of tryptophan degradation and its properties in this snail model using extracts from naturally occurring plants such as sage, lemon balm, and hawthorn berry.

## Figures and Tables

**Table 1:** Experimental design for an in vivo pharmacological study.

Number of Groups/Name	Treatment
Group 1: CO1	Water
Group 2: CO2	Water
Group 3: L-KYN	L-kynurenine
Group 4: D-CYC	D-cycloserine
Group 5: CER	Cerebrolysin
Group 6: L-KYN/D-CYC	L-kynurenine + D-cycloserine
Group 7: L-KYN/CER	L-kynureniene + cerebrolysin

Abbreviations: Control (CO); L-kynurenine (L-KYN); D-cycloserine (D-CYC); Cerebrolysin (CER).

**Table 2:** **Statistical analysis.** (**a**). Student’s *t*-test of significance for KAT I. (**b**). Student’s *t*-test of significance for KAT II. (**c**). Student’s *t*-test of significance for KAT III; ns—not significant.

**(a)**			
**Treatment**	**1 h–2 h**	**1 h–4 h**	**2 h–4 h**
CO	ns	0.001	0.009
Cer 0.25 µL	0.008	0.021	ns
Cer 2.5 µL	ns	0.012	0.029
D-Cyc 200 µM	ns	0.03	ns
D-Cyc 400 µM	ns	0.018	0.048
L-Kyn 200 µM	ns	ns	ns
L-Kyn 400 µM	ns	0.001	0.003
**(b)**			
**Treatment**	**1 h–2 h**	**1 h–4 h**	**2 h–4 h**
CO	0.007	0.004	0.007
Cer 0.25 µL	ns	ns	0.049
Cer 2.5 µL	ns	ns	ns
D-Cyc 200 µM	ns	0.004	0.013
D-Cyc 400 µM	0.016	0.041	ns
L-Kyn 200 µM	0.030	0.001	0.002
L-Kyn 400 µM	0.002	<0.001	<0.001
**(c)**			
**Treatment**	**1 h–2 h**	**1 h–4 h**	**2 h–4 h**
CO	0.034	<0.001	<0.001
Cer 0.25 µL	ns	ns	ns
Cer 2.5 µL	ns	ns	ns
D-Cyc 200 µM	ns	0.044	ns
D-Cyc 400 µM	ns	ns	0.023
L-Kyn 200 µM	0.038	<0.001	0.001
L-Kyn 400 µM	0.027	0.001	0.004

## Data Availability

All relevant data are available from the corresponding author upon reasonable request.

## Abbreviations

AMPOL—2-amino-2-methyl-1-propanol; ANA—anthranilic acid; ANOVA—analysis of variances; ARS—age rating scale; Cer—Cerebrolisin; D-cyc—D-cycloserine; EAA—excitatory amino acid; nAChR—nicotinic-acetylcholine receptor; CG—cerebral ganglia; CNS—central nervous system; CS—conditioned stimulus; CSF—cerebro spinal fluid; GDF—glia depressing factor; GPR35—G protein-coupled receptor 35; HPLC—high performance liquid chromatography; KAT—kynurenine aminotransferase; KYNA—kynurenic acid; L-KYN—L-kynurenine; N—number; NMDA—N-methyl-D-aspartate; P_5_P—pyridoxal 5′-phosphate; PYR—pyruvate; SG—subpharyngeal ganglia; TCA—trichloroacetic acid; TRP—tryptophan; US—unconditioned stimulus; XAN—xanthurenic acid.
